# Supplementary material for: Are carbon emissions trading and green financial instruments synergistic? -Comprehensive quantitative research based on content analysis
Source: PLoS One. 2024 Mar 7;19(3):e0298601. doi: 10.1371/journal.pone.0298601 (PMC10919601; doi:10.1371/journal.pone.0298601)
Supplement: S1 Data — (ZIP) [file pone.0298601.s001.zip › Data/地级市Z_F工作报告.zip!/║ú─╧/║ú┐┌╩╨/└┤╫╘╣1⁄2╓┌║┼í╛┬φ┐╦╩2╛▌═°í┐_2012.docx]

各位代表：

现在，我代表市人民政府向大会报告工作，请予审议，并请各位政协委员和其他列席人员提出意见。

一、２０１１年和本届政府工作回顾

过去的一年，在市委的坚强领导下，我们始终以科学发展观为指导，积极应对复杂的经济形势，奋力拼搏，攻坚克难，较好地完成市十四届人大六次会议确定的年度目标任务，实现“十二五”良好开局。实现地区生产总值７１２．７５亿元，增长１２．３％；财政总收入１８８．８２亿元、增长１５．３％，地方一般预算收入６０．９３亿元、增长２１％；固定资产投资４０４．６亿元，增长２６．２％；社会消费品零售总额３８７．１８亿元，增长１８．１％；城镇居民人均可支配收入１９７３０元、增长１８％，农民人均纯收入７１６０元、增长１６％；居民消费价格涨幅５．４％；城镇登记失业率０．９４％；人口自然增长率８．２‰；全面完成省下达的减排任务。

（一）科学研判宏观形势，采取得力措施，着力提高政府应对复杂局面的能力和水平。一是按照市委“十二五”规划建议，编制“十二五”规划纲要和１６个重点专项规划，储备重大项目，进一步细化、充实和完善未来五年的规划体系。二是实施重点产业发展扶持政策，出台加快工业发展的若干规定以及鼓励航空、会展、金融和资本市场、总部经济、文化产业、航运发展等优惠政策。积极扶持中小企业发展，为７００多家企业提供融资贷款２７亿元。三是开工建设保障性住房１１７０２套，改造农村危房２０３６户，分别占省下达任务的１４７％和１００％。探索廉租房和公租房、限价商品房和经济适用房“两个并轨”。严格落实国家楼市调控政策，调整房地产开发结构，产权式酒店管理规定在全国具有开创性。四是狠抓项目建设，２５个省重点项目和５５个市重点项目顺利推进，竣工３３个超额完成年度投资计划。五是多措并举稳定物价，有效解决猪肉市场价格上涨过快问题，实施“菜篮子”工程，扶持蔬菜生产、流通、储备和农超对接，建立蔬菜基地７０４８亩，推出一批平价菜超市和农产品直通车。物价涨幅低于全省平均水平。六是取得抗击强台风强降雨重大自然灾害的全面胜利。

（二）谋大局、抓大事，扎实推进重大项目和重点工作，为“十二五”规划的全面实施奠定基础。一是精心谋划和积极争取重大项目。全面启动“十二五”十大攻坚战。南渡江流域３０万亩土地整治、南渡江引水工程、南渡江左岸堤防工程、现代农业示范区、“金盾”工程、城市防灾减灾体系能力建设等重大项目列入国家和省“十二五”规划。举办海口“十二五”规划纲要暨重大投资项目发布会，与中石油、大唐电信等企业签订战略合作协议，与中交集团合作推进港区建设。二是着力构建特色现代产业体系。大力发展设施农业、生态循环农业、特色休闲农业，积极推进传统农业向现代农业转变。冬种瓜菜面积达１８．９万亩，新增特色花卉基地４５００亩，建成２个国家水产健康养殖示范点、１２个标准化养殖小区，建成７个大中型、联户型沼气池，创建休闲农业示范点１５个，新增造林１．６万亩。加强农业基础设施建设，１０座小型水库除险加固和２８栋防洪楼建设全面启动，防洪防潮工程加快推进。实现农业总产值７６．８亿元，增长６．６％。努力提升工业规模和质量。机电制造、化工化纤纺织、食品和农产品加工等传统产业巩固壮大，海马汽车销售总额超过百亿元，３０万箱红塔卷烟易地技改、椰岛保健酒技改等骨干项目加快推进。以新能源、医药制造为重点的战略性新兴产业加快发展，英利二期前２００兆瓦建成投产，“海口药谷”新增入园企业１３家。筹划布局５０平方公里美安科技新城，海口综合保税区封关运行。实现工业总产值５３９．３３亿元，增长１４．７％。以旅游业转型升级、现代物流、金融保险为重点，加快发展现代服务业。建成海南国际会展中心，海口船舶游艇交易所正式挂牌，新建游艇泊位１４０个。成功举办高尔夫世界杯、“海口之春”旅游文化艺术节等重大活动８０多个。完成海南国际创意港一期。全国首家机场离岛免税店正式营业，美兰机场进出岛旅客突破１０００万人次。实现旅游总收入８３．０２亿元，增长１５．２％。马村港二期和新海港建设顺利推进，海南中商农产品中心市场投入使用，海南国家级水产物流交易中心主体工程基本完工，现代物流平台正在搭建。海口港运送旅客突破８５０万人次，货物吞吐量突破４３００万吨、集装箱突破８０万标箱。海口农商银行正式成立，引进２家股权投资基金，协调１家保险公司和１家大型财务公司落户，中信银行、招商银行、民生银行获批在海口设立分行。实现第三产业增加值４８６．６８亿元，增长１２．４％。三是以规划为龙头，狠抓城市建设与管理。完成２７个片区控规，主城区控规基本实现全覆盖。编制“十二五”棚户区改造规划，完成海甸溪北岸、镇海村安置房建设，完成朱云片区、滨江新城起步区拆迁征收工作。新埠岛起步区、西海岸南片区、金沙湾片区开发建设顺利推进。建成１１０公里绿色慢行系统。完成滨海大道示范段和滨海西路南侧园林景观改造。丘海大道延长线正式通车，海甸岛环岛路、滨海西路二期、龙昆南路延长线等顺利完工，南海大道整治按进度推进。继续实施“五大工程”，“打违”工作力度空前，全年共拆除违法建筑５７２宗６９．７万平方米。

（三）直面改革与发展中存在的突出问题，深化改革开放，努力增强推动科学发展的内生动力。一是继续深化行政管理体制改革。落实市委关于进一步强区扩权的决定，再次向区级下放行政权项３４项，调整市区两级在人力、财政、土地收益等方面的资源配置。深化一般预算资金支出和部门预算审批管理改革。二是大力推进投融资体制改革。组建海口公交集团，设立城市发展基金，参与筹建大宗商品期货交易中心，引入省政府和海南钢铁公司注资市担保公司，筹划建立海口金融区。三是用足用好国家扶持政策。积极争取国家和省的支持，国土资源部在支持海南国际旅游岛建设２０项政策措施中单独增加对我市的扶持措施，省政府对我市常年瓜菜基地建设加大资金支持，大唐电信在我市设立２．５亿元的信息产业创业投资基金等。积极解决观澜湖项目用地等问题。四是推进社会事业改革。完成基层医疗卫生机构综合改革，率先在全省农村卫生室实施国家基本药物制度。推进事业单位人事制度改革，新进人员全部实行公开招聘。扩大政府购买服务范围，引入社会组织参与审计、资产评估等工作。五是着力扩大对外开放。组织参加第七届泛珠大会等一系列重大区域性经贸活动。召开第二届海外智囊团年会，新增２个国际友好城市。被确定为唯一代表我国申办２０１７年世界石油大会的城市。

（四）用情用心用力改善民生，着力解决群众关注的问题，让市民更多地共享改革发展成果。新增财力７０％投向民生领域。扎实开展国家创业型城市创建，投入５２７９万元落实各项就业政策，发放创业小额担保贷款３１００万元，启用新的人力资源市场，城镇新增就业人员４万人，农村富余劳动力转移就业１．４３万人。大力落实公交优先战略，白沙门、长流起步区公交首末站功能性投入使用，建成公交候车亭９９座，优化调整公交线路１３条，投放节能环保及新能源公交车２３０辆，新增出租车３００辆，更新出租车５１６辆，全市小学生实现免费乘坐公交车。在全省率先实施“文明大行动”，集中开展“建设国际旅游岛，安全畅通文明行”活动，实施城市道路交通联席会议制度，出台电动车管理办法，大力整治“三车”，城市交通秩序明显改善。实际投资8．8亿元，打通侨中路、高登东路、和平南路、文明东路、世贸东路延长线五条“断头路”，新建４座人行天桥，有效缓解国贸、海秀、龙昆南等路段交通压力。拉平市与省公务员收入差距，增加环卫、园林、居委会退休人员等较低收入公共服务人群的收入。中小学校安工程三年计划项目全部开工，全面启动８所标准化公办幼儿园建设。完成旅游医疗服务保障中心，改扩建社区卫生服务中心４家，农村卫生室标准化率达７０％。企业人均养老金提高到１４１０元，新型农村合作医疗筹资标准提高到２８０元／人，城镇居民医保重大疾病医疗费最高报销限额提高到２０万元，普通门诊报销比例提高到５０％。开展城镇居民养老保险试点工作。筹资１．２亿元，救助城市低保和农村低保对象５．８万人次，改扩建敬老院４家。投入扶贫资金４３３４万元，贫困村庄和革命老区８万多人受益。开展市领导联合大接访活动，接待信访群众１３２批次。抓好矛盾纠纷排查调处工作，化解矛盾纠纷４８７２起。办理法律援助案件６０１８宗。开展打击“两抢一盗”、“打黑除恶”、“清网”等专项整治行动，命案破获率居全国省会城市前列，社会治安持续好转。安全生产形势保持稳定。食品药品、口岸、统计、气象、人防、档案、史志、外事侨务、民族宗教等工作取得新成绩，工会、妇女、儿童、青少年、老龄、残疾人、慈善等事业取得新进步。

（五）坚持用制度管人管事，加强执行力建设努力建设法治、民本、高效、廉洁政府。落实重大事项向市委报告制度，定期向市人大常委会报告、向市政协通报工作，畅通政府与市委、市人大常委会、市政协的经常性沟通渠道。人大议案、政协提案办复率１００％。制定规范性文件３８个。依法行政工作连续三年全省考核第一。建立网络问政平台。出台资源优先配置、环境监督登记等重点项目推进十项制度，对工业、旅游等“三类”建设项目实施联合审批。完善政府工作规则和督办办法，机关办文办会办事水平得到提高。开展公务车专项清理。强化行政监察和审计监察，完善电子监察系统督办平台，政府廉政建设取得新成效。

２０１１年取得的这些成绩，是在历届政府班子和本届政府前几年打下的良好基础上取得的。市十四届人大一次会议以来的五年，是海口经济社会发展最好、民生投入最多、改革开放力度最大的时期，在全省的龙头引领地位进一步增强，省会中心城市功能不断完善。五年来，我们牢牢把握发展第一要务，积极推进经济结构优化升级，经济发展上了一个很大台阶。高度重视产业的发展和培育，以南渡江流域３０万亩土地整治为代表的现代农业和城乡统筹已经破题，以英利为代表的新能源工业开始崛起，以观澜湖为代表的高端旅游业开始出现，以国际会展中心为代表的会展业开始形成。地区生产总值年均增长１２．５％，实现翻番；财政总收入年均增长２１．９％，增长１．７倍；固定资产投资年均增长２２．８％，增长１．６倍。五年来，我们狠抓基础设施建设，大力推进城市化和城乡一体化，城乡面貌发生深刻变化。城市建成区面积五年增长３８．３％，城镇化率达到７４％。“六纵一横”的对外交通公路体系和“八纵六横”的市区主干道网基本形成。棚户区改造与新区开发成效明显，城市环境综合整治常态化、长效化。人均公共绿地达１２平方米，森林覆盖率达３８．４％。城镇生活垃圾无害化处理率达１００％，城市污水处理率达８７．４％。生态环境始终保持全国一流。五年来，我们坚持深化改革开放，不断创新体制机制，发展的动力和活力明显增强。深化政府行政管理体制改革，实施两轮强区扩权，完成新一轮政府机构改革，推进行政审批大提速，行政效能显著提高；财政体制、投融资体制、国有企业、社会事业、林权制度和农村综合改革取得重大进展，海口的知名度和影响力进一步扩大。五年来，我们注重统筹兼顾，加快推进各项民生社会事业，和谐社会建设扎实推进。荣获“国家创新型试点城市”；基础教育均衡发展，职业教育迈上新台阶，新增义务教育和高中学位３万个；市、区、镇（街道）、村（居）四级公共文化服务体系基本建立，群众文化生活进一步丰富；医疗卫生水平明显提升。城镇居民人均可支配收入和农民人均纯收入五年分别增长８４．２％和７２．４％。新增就业２４万人次，连续五年实现零就业家庭动态为零。社会保障制度实现全覆盖，农村医保、职工医保、城镇居民医保和最低生活保障标准大幅提高。开工建设４３１３２套保障性住房，超额完成省下达的任务。市民素质和城市文明程度明显提升，获得“全国文明城市”提名资格。

各位代表，回顾过去五年的辉煌历程，我们深切地感到，之所以能够在复杂多变的形势中，乘势奋进，实现又好又快发展，这是市委科学决策、坚强领导的结果，是市人大及其常委会和市政协支持监督的结果，凝聚着全市广大干部群众和社会各界的心血和汗水，充分展示了海口人民团结拼搏、一往无前、奋发图强的决心、勇气和智慧。在此，我代表市人民政府，向全市人民，向人大代表、政协委员，向各民主党派、各人民团体，向驻市单位、驻市部队、武警官兵和公安司法干警，向所有关心和支持海口建设的社会各界人士，一并表示衷心的感谢！

在看到成绩的同时，我们也清醒地认识到还存在一些突出问题：欠发达的基本市情没有根本改变，综合竞争力不强，抗风险能力较弱，加快发展和产业结构转型升级的任务十分繁重；城市功能不够完善，管理水平有待提高，中心镇建设滞后，省会中心城市的辐射带动作用没有充分发挥出来；经济社会转型中矛盾交织多变，统筹发展的难度加大；历史欠账较多，居民生活水平还比较低，保障和改善民生的任务仍然紧迫艰巨；体制机制创新和对外开放的空间还比较大，与建设国际旅游岛的要求尚有较大差距；有些部门及公务人员的思想观念、服务意识、工作作风、办事效率和执行能力尚不适应工作需要，整体素质亟待提高，等等。对此，我们必须高度重视，在今后的工作中采取有力措施，切实加以解决。

二、今后五年的总体思路和２０１２年工作重点

根据省“两会”和市第十二次党代会精神，今后五年政府工作的总体思路是：以科学发展观为指导，紧紧抓住国际旅游岛建设重大机遇，牢牢把握经济建设这个中心不动摇，以转变发展方式为主线以创新体制机制为动力，以保障和改善民生为根本，致力于加快城市转型，致力于发展实体经济，致力于强化省会城市功能，建设全市人民幸福家园，努力打造“宜居、宜业、宜学、宜游”最精最美的省会城市。

未来五年，我们必须更加注重实体经济，坚持以新型工业化带动产业结构的调整，以健全的现代产业体系支撑经济结构的优化和发展方式的转变，强化省会中心城市经济极核地位，在国际旅游岛和环北部湾区域经济发展格局中发挥更大的辐射作用。必须更加注重城乡统筹，下决心完善城市规划，加强城市管理，以城市重点片区的开发建设带动城乡发展，完善城乡基础设施，推进城乡公共服务均等化，促进城乡共同繁荣，建设功能完善的现代化滨海城市。必须更加注重文化建设，坚持科教和人才兴市战略，充分发挥国家历史文化名城资源优势，加快人文资源优势向旅游经济优势转变，推进文化大发展大繁荣，着力提升城市文化软实力，打造国际旅游岛文化高地。必须更加注重民生事业，创新和加强社会建设与管理，千方百计提高居民收入，以办好“润物细无声”的实事好事，赢得人民群众的理解、支持和拥护，促进社会和谐进步。必须更加注重改革开放，直面改革发展中的突出问题，以更大的胆略、更广的视野、更前瞻的思虑谋划好科学发展的路数，当好全省改革创新和对外开放的排头兵。必须更加注重生态文明，坚持生态立市，在保护中开发、开发中保护，严格执行环保准入制，全面落实节能减排等约束性指标，生态环境始终保持一流。

未来五年，我们最重要的任务是全力贯彻落实好海口市“十二五”国民经济和社会发展规划纲要，一张蓝图绘到底，负重奋进促转型，坚持不懈抓落实，抓住机遇快发展。２０１２年是新一届政府的起始之年，做好全年工作，必须始终把保持经济较快增长作为刚性任务，把扩大投资特别是扩大社会投资作为着力重点，把消费结构调整作为扩大消费和调整投资的重要导向，把全面优化升级产业结构作为政府主导发展的基本思路，把改革开放、完善政策体系作为掌握发展主动权的牵引抓手，把改善和保障民生作为根本目的，切实把“稳中求进”和“逆势上扬”的总基调贯穿到各项工作部署和具体要求中。全年经济社会发展的主要预期目标是：地区生产总值增长１３％左右，全市一般预算收入和地方一般预算收入均增长１５％左右，全社会固定资产投资增长２８％左右，社会消费品零售总额增长１９％左右，城镇居民人均可支配收入和农民人均纯收入均增长１３％左右，全面完成省下达的节能环保指标和物价调控指标。

为实现今年目标，要重点抓好十个方面的工作：

（一）扎实开展“项目建设年”，全面提升经济管理水平。按照市委确定的发展理念和城市定位，着眼于完善省会城市功能区划，调整和修编城市总体规划、土地利用规划和基础设施规划，以城市空间布局和产业布局为引导，以空间换时间、资源换项目，积极引导各类优质资源向重大项目、重点产业、重点领域集中，确保重点项目“建成投产一批、开工建设一批、谋划储备一批”。一是加大投资力度。把产业强市作为海口当前发展的第一要务，突出抓好２１个“十大攻坚战”项目和６４个市重点项目，力争全社会固定资产投资突破５００亿元，确保重大基础设施建设项目、重大城市功能完善项目、重大民生项目按时间节点开工竣工。英利二期、红塔技改、汉能一期等项目要如期投产，海马三期、狮子岭二期启动征地工作，东海岸人工岛、新海物流园、新海港区汽车客运滚装码头一期、兰桂坊、长影海南世纪影城、东寨港红树林国家湿地公园、热带野生动植物园新区等一批项目要取得实质进展。积极争取政策支持，力争一批重大项目纳入国家和省的规划。在招商引资上项目、政策优惠引项目方面加大力度，成立专业招商团队，完善激励机制，充分激活社会投资。二是做好前期工作。把更多的精力放在项目的前期工作上，在策划包装、勘测设计、资金筹措、土地供应、拆迁安置、人才储备、开发模式等方面做实做细。三是强化推进机制。加强质量和标准管理，完善项目评估评价体系，落实“六个一”责任制，提高项目组织化水平，确保重点项目不超概算、不超工期、不发生质量事故、不发生安全责任事故、不引发重大群体性事件、不倒干部，形成“抓一个项目，成一个亮点，铸一个品牌”的良性机制。

（二）实施工业优先战略，推进工业提速发展。坚持工业向园区集中、园区向西部转移，继续推进“两区八园”功能完善和升级改造，形成综合保税区和高新区“前店后厂”发展格局，力争两区增加值分别增长２０％和５０％以上。完成美安科技新城产业规划，明确产业定位，加快推进１５平方公里起步区建设。投入扶持资金２亿元，落实加快工业发展的各项政策。强化技术创新的支撑作用，着力改造提升机电制造、化工化纤纺织、食品和农产品加工三大传统优势产业。依托海马、英利等骨干企业和“海口药谷”，积极培育壮大新能源汽车、新能源、生物医药、新一代信息技术等战略性新兴产业。新增高新技术企业８家以上。选择１—２个镇作为试点，把产业引入镇域经济中，打造特色专业加工镇。完善适应中小企业特点的担保融资体系，推动中小企业提高协作配套发展水平。力争工业增加值增长１７％以上。

（三）以现代服务业为重点，提升第三产业水平。大力开展“城市营销年”活动，精心组织高尔夫、游艇经济两大论坛和“海口之春”旅游艺术节等一批重大活动，唱响“阳光海口、娱乐之都、品位之城”主题。出台新的旅行社奖励政策，鼓励旅行社积极开拓市场。突出机场离岛免税特色，完善配套服务，推进旅游商品研发中心、交易中心建设，鼓励和引导专业商品市场集聚发展，着力把海口打造成为全省和环北部湾区域购物中心。成立专门机构，积极引进和培育一批高水准的会展专业服务企业。力争社会消费品零售总额增长１９％以上，旅游总收入增长１２％以上。发挥综合保税区物流中心功能，推动大型物流仓储配送中心、口岸物流平台建设，完善城市物流配送体系，加快发展现代物流业。打造专业化团队，兑现扶持政策，优化金融生态环境，引进金融企业３家以上。积极调整房地产结构，促进房地产业健康发展。加快服务行业规范化、标准化建设，强化行业协会管理和从业人员培训，推动诚信体系建设。力争第三产业增加值增长１２％以上。

（四）围绕现代农业发展，扎实推进新农村建设。坚持科学规划引领、富民产业支撑、基础设施推动，进一步完善新农村建设推进机制。制定海口市城乡经济社会统筹发展规划，积极申报国家城乡统筹综合改革试点城市。坚持以基础设施建设适度先行推进公共服务向农村延伸，以城市的功能完善和布局调整带动农村产业培育和专业化发展，以战略性新型产业特别是加工业的发展，促进农业的规模化、集约化经营，以第三产业特别是现代服务业的快速增长为务工农民提供更多就业岗位，着力增加农民经营性、工资性、财产性收入。按照“五大示范基地、一大改革示范区”目标，基本完成南渡江流域土地整治一期工程。基本完成罗牛山农产品加工产业园一期建设，启动泉溢２．５万吨水海产品综合加工项目和１０里花卉长廊建设。建成４个农产品冷藏保鲜库和１７个镇级农村推广服务站，建立健全市、区、镇、村农产品质量安全检测监管体系和农产品流通体系。强化科技对农业的支撑，建好２０个农业科技１１０、电子农务服务点；实施“阳光工程”培训计划，培训农村劳动力１．２万人，转移输出农村劳动力１万人。做好南渡江引水工程前期工作，加快推进龙塘大坝至东山段防洪堤和农村排涝工程，继续改造农村水网、电网、路网、气网和信息通讯网，进一步提高农业综合生产能力。启动计划单列镇试点，扶持发展非农经济，推动镇域经济加快发展。全面启动并完成５０％的村庄规划编制。出台鼓励社会资本投资发展农村经济的政策。继续开展镇村环境综合整治，打造一批旅游风情小镇和文明生态村。以畜禽养殖污染治理为重点，控制农业面源污染，优化农村生态环境。适当提高农村“五保五险”水平，扩大覆盖面。加强农村文化建设，积极引导村风民俗文明化，提高农村文明水平。加大扶持力度，全面创新和提升农村社会事业的管理服务水平。深入开展“绿化宝岛”行动，加强生态公益林和景观林建设，提升改造海防林，完成造林１．５万亩。力争农业增加值增长８％以上。

（五）完善中心城市功能，进一步提升城市品位。紧扣城市历史脉承、文明延续、文化底蕴、风土人情，进一步完善城市规划体系，引领建设具有独特产业体系、独特文化品质、独特风貌品牌、独特生态环境、独立精神内涵的“四宜”最精最美城市。完成城市综合防灾规划等５个专项规划编制。加快立体交通体系建设，启动城市轨道交通、海秀路高架桥项目，积极推进美兰机场扩建。大力实施“以港兴市”战略，积极推进与中交集团合作，同步建设马村港三、四期，加快秀英港搬迁步伐。健全大交通体制，完善城市路网，开展“９＋１再行动”，海府一横路延长线、美祥东路贯通工程、绿色长廊二期、高登西路等９条断头路实现功能性通车，完成红城湖路延长线工程的全部拆迁安置任务。博义、夏瑶、丁村等城中村改造必须全面启动，落实业主。西海岸南片区建设上半年正式开工。积极推动江东开发建设。完成白沙门污水厂一期脱氮除磷改迁项目。抓好沿海防潮工程和市区主要积水点的改造。加强城市精细化管理，逐个破解社会管理难题。以第八届泛珠大会为契机，完成空港、海港和高速公路出入口关键部位和节点的环境整治。完善大城管体制，在行政执法、市政设施、园林绿化、环境卫生等方面形成更大合力，深入开展城市环境综合整治“五大工程”。巩固扩大“打违”成果，确保“零增量、减存量”。坚持有载体、有抓手、有特色，扎实开展“文明大行动”，继续推进创建国家卫生城市活动。

（六）不断深化改革开放，增强科学发展活力。推进城市建设投融资体制改革，完善政府投资项目代建制。规范地方政府性债务管理，设立偿债准备金专户，防范地方政府债务风险。加快财税体制改革，增强区级发展主动性。积极解决琼山历史遗留问题。加快土地管理制度改革，建立土地利用总体规划实施评价体系，启动农村土地产权制度改革和“三旧”改造试点，积极盘活存量用地和闲置用地。加快国有资产战略重组。深化审批制度改革，尽快出台优化流程、减少审批程序、实行并联审批的行政决定。出台鼓励民营企业发展实体经济的政策。稳步推进公立医院改革，全面实施国家基本药物制度。完善政府购买公共服务机制，培育壮大社会中介组织。推进行政事业单位收入分配制度改革。在游艇水域和低空空域开放、竞猜型和即开型彩票等国际旅游岛特殊政策的落实上争取先行先试。积极推动马村港区列入国家汽车整车进口口岸。举全市之力做好申办世界能源大会和世界石油大会工作。加强与琼北市县合作，加快构建省会经济圈。力争实际利用外资增长１０％以上，实现出口总值增长１２％以上。

 (七)促进文化旅游融合发展，提升城市软实力。认真落实市委《关于加快文化产业发展若干政策措施的意见》。设立１０００万元的文化产业发展专项资金。全面启动五公祠、海瑞墓、丘浚墓三大人文景点的改造提升，调整完善规划，完成大部分征地任务；探索创新推进模式，骑楼建筑历史文化街区保护与综合整治工程要取得实质性、有形象的进展，为三年内全部完成任务奠定坚实基础。启动五源河文化体育中心项目建设。鼓励地方特色旅游商品的市场开发，培育发展广告设计、演艺娱乐、设计服务、影视制作等创意产业。鼓励社会资本投资旅游演艺产业，扶持民间艺人组建文艺表演团体，推出一批游客和市民喜闻乐见的旅游演艺品牌。

（八）创新和加强社会管理，促进社会和谐。密切关注新情况新问题，把社会管理放在与经济发展同等地位来抓，以项目为载体，以规划为核心，以社区为基本，实施网格状管理，让社会变得更加有序，给群众提供方便、安全、文明的生活环境。落实社会管理的责任主体，明确市、区、镇（街道）、村（社区）职责和分工，努力形成事事有人管、出事有人负责的工作格局。做好群众工作，加强舆情分析，高度重视网络民意，完善常态化的网络问政机制。扎实做好信访工作，完善“大调解”机制，切实把矛盾解决在基层，化解在萌芽状态。加强社会管理综合治理，强化实有人口管理，推进城市监控与报警系统等公共安全项目建设，预防和打击各类违法犯罪活动。规范和加强城乡社区建设，把更多的资源投向基层，提升管理服务水平，打造一批文明和谐社区。突出抓好旅游、交通、施工、建筑消防和食品药品安全监管，坚决遏制重特大事故发生。强化对自然灾害、突发事件的预防、监控和处置，提高城市应急管理能力。加强人口与计划生育工作，稳定低生育水平，统筹解决人口问题。充分发挥工会、共青团、妇联等人民团体以及社会组织在参与社会事务管理中的作用。推进“双拥”共建，巩固军政军民团结。继续做好统计、气象、人防、档案、史志、老龄、外事侨务、民族宗教等工作。

（九）加强政府自身建设，打造人民满意政府。自觉接受市人大及其常委会的法律监督和市政协的民主监督，主动接受社会监督和舆论监督。继续深入推进法治、民本、高效、廉洁政府建设，大力开展“效能建设年”活动。围绕服务企业、服务项目、服务基层、服务群众，切实改善政务环境，转变机关作风。各区各部门要逐级为每一个岗位量身制定科学具体的职位说明书，细化目标、量化责任，切实提高行政执行力。严格依法行政，开展相对集中行政复议权和行政复议委员会试点工作。加强政府信息化建设，进一步扩大政务信息公开范围。更加注重反腐倡廉建设，强化行政监察和审计监督，抓好中央、省、市重大决策部署落实情况和重大支出项目的专项监督检查，切实解决损害群众利益的突出问题。完善绩效考核体系，强化激励约束，真正奖勤罚懒。广大公务人员要切实增强责任心、爱心、公心，拼搏实干、廉洁自律，真正担当起历史赋予我们的使命。坚持维护正义、弘扬正气、鼓励正直，更好地在全市树立实干之风、为民之风、进取之风、清正之风！

（十）办好“十大民生实事”，建设百姓的幸福家园。一是完善城乡统筹创业、就业扶持政策，加强创业孵化基地和创业项目库建设，提高职业技能培训水平，城镇新增就业３．５万人以上，城镇登记失业率控制在３％以内。二是保质保量按时完成省下达的保障性住房建设任务，完善已建保障性住房小区周边路网和基础配套设施；对已售但未办产权证住房，开展集中专项清理办证行动。三是继续加强交通环境综合整治，优化重点片区交通组织，积极缓解交通拥堵；优化和新增一批公交线路，新投放公交车２００辆、出租车２８０辆，新建一批公交候车亭，开工建设６个公交场站。四是切实抓好“菜篮子”工程，新建１万亩常年蔬菜基地、４个冬季瓜菜生产示范工程和１５个标准化畜禽生态养殖小区，新开设平价菜超市、商店２０家以上，规范降低集贸市场摊位费和超市进场费。五是抓好城乡低保规范化和动态管理，做到应保尽保、分类施保；新改扩建敬老院６家，进一步提高农村“五保”集中供养率；大幅提高扶贫标准。六是加强社会养老服务体系建设，城区社区居家养老实现全覆盖，对８０岁以上高龄老人发放长寿补贴；对困难群众基本殡葬服务费用实施减免，抓好公益性公墓建设。七是实施学前教育助学券制度，新改扩建公办幼儿园１６所；积极解决城区“麻雀”学校问题，撤并、整合一批小规模学校，推动农村寄宿制学校和乡镇中心学校建设；高度重视校园安全，完成校舍安全工程，完善校车管理与运营模式。八是提高基层卫生服务水平，城镇居民规范化电子健康档案建档完成７０％以上，新建６４家农村标准化卫生室，村卫生室标准化率达１００％，为城乡居民免费提供基本公共卫生服务项目和重大公共卫生项目。九是丰富城乡群众文化生活，启动四个区文化馆建设,组织开展各类文化体育活动４０００场次和文化下乡7０００场次以上。十是完善城乡商业经营服务网络，改造农贸市场２７家，新建城乡连锁超市３家、农资农家店２０家。在此基础上，及时解决群众反映强烈的新问题，量力满足群众期盼的新需求。

各位代表，过去的五年，我们经受并战胜了各种严峻考验，更加坚定了我们勇往直前的信心和决心。把海口建设得更加美好，是２０９万椰城人民的期待和重托。让我们在市委的坚强领导下，以更加昂扬的斗志、更加务实的作风，奋力推进海口在国际旅游岛建设的大潮中凭风借力、扬帆远航，以优异成绩迎接党的十八大和省第六次党代会胜利召开！
